# Establishing a faith-based organisation nursing school within a national primary health care programme in rural Tanzania: an auto-ethnographic case study

**DOI:** 10.3402/gha.v9.29404

**Published:** 2016-05-27

**Authors:** Alexander Bischoff

**Affiliations:** 1 Division of Tropical and Humanitarian Medicine, University Hospitals of Geneva, Geneva, Switzerland; 2 School of Health Sciences, University of Applied Sciences and Arts Western Switzerland, Fribourg, Switzerland

**Keywords:** international collaboration, primary health care, human resources for health, nursing

## Abstract

**Background:**

In 2007, the Tanzanian government called for improvements in its primary health care services. Part of this initiative was to accelerate the training rate for nurses qualified to work in rural areas. The aim of this study was to reflect on the issues experienced whilst establishing and implementing a faith-based organisation (FBO) nursing school and make recommendations for other similar initiatives.

**Design:**

This paper describes an auto-ethnographic case study design to identify the key difficulties involved with establishing and implementing a new nursing school, and which factors helped the project achieve its goals.

**Results:**

Six themes emerged from the experiences that shaped the course of the project: 1) Motivation can be sustained if the rationale of the project is in line with its aims. Indeed, the project's primary health care focus was to strengthen the nursing workforce and build a public–private partnership with an FBO. All these were strengths, which helped in the midst of all the uncertainties. 2) Communication was an important and often underrated factor for all types of development projects. 3) Managing the unknown and 4) managing expectations characterised the project inception. Almost all themes had to do with 5) handling conflicts. With so many participants having their own agendas, tensions were unavoidable. A final theme was 6) the need to adjust to ever-changing targets.

**Conclusions:**

This retrospective auto-ethnographic manuscript serves as a small-scale case study, to illustrate how issues that can be generalised to other settings can be deconstructed to demonstrate how they influence health development projects in developing countries. From this narrative of experiences, key recommendations include the following: 1) Find the right ratio of stakeholders, participants, and agendas, and do not overload the project; 2) Be alert and communicate as much as possible with staff and do not ignore issues hoping they will solve themselves; 3) Think flexibly and do not stubbornly stick to original plans that might not be working; 4) Be realistic and do not romanticise. Embarking on such a project was a timely response to the Tanzanian's government call for strengthening Primary Health Care and for rapidly accelerating the training of nurses able to work in rural areas.

## Introduction

In 2006, three major topics dominated the globalisation and health debate worldwide at the time: the renewal of primary health care worldwide; the workforce crisis in general, and the lack of nurses in particular; and public–private partnerships (1). These issues were particularly pertinent in Tanzania. The Tanzanian government devised a primary health services development programme, and it focused on three main areas that needed improvement: 1) strengthening the health systems, 2) rehabilitation, and 3) human resource development (2). Its aim was to have a dispensary in every village, a health centre in every ward, and a district hospital in every district. The implications of these policies meant that more nurses were needed; this required not only training far more nurses than before, but also training them more quickly.

The projections given by the Tanzanian Ministry of Health and Social Welfare, in 2007, showed that the number of registered nurses required was 20,000, but at that time, only slightly more than 3,000 were working. The gap, therefore, meant that 17,000 nurses had to be trained, necessitating the construction of 18 new nursing schools to meet the demand for training sufficient numbers of qualified nurses (2). The 2008 Tanzanian report on Human Resources for Health summarised the two main objectives to 1) upgrade and establish more training institutions to ensure adequate availability of skilled human resources for health care and 2) fast-track the capacity building and upgrading of allied health workers to meet the needs of the primary health care facilities (3).

The ambitious goal set by the Primary Health Services Development Program could only be achieved by the contribution of public–private partnerships. In Tanzania, these partnerships are mostly provided by faith-based organisations (FBOs). In 2008, the Tanzanian government issued the following statement: ‘The Government of Tanzania is promoting the concept of Public Private Partnership (PPP) in which the Government through the Ministry of Health and Social Welfare provides grants in aid to the Faith-based Organisations according to the contract service agreements dependent on the number of hospital beds. Similarly, the government provides aid grants to the training institutions according to the school student capacity as per training agreements’ (3).

The United Nations Populations Fund defines FBOs ‘as Faith-based or faith-inspired non-governmental organisations, with legal standing, which are working to advocate for, or deliver development and humanitarian services whether nationally, regionally, or internationally’ (4: 22). Despite the general lack of empirical data, the same authors conclude that FBOs are important actors in Sub-Saharan health systems. ‘FBOs deliver a substantial volume of health care, and their common visions of stewardship, inclusiveness, dignity, and justice make many such organisations ideally suited as key partners for delivering the post-2015 Sustainable Development Goals’ (5: 1).

FBOs have a long tradition of providing health care in Africa, and 30–70% of all healthcare provision and education is provided by FBOs (6). In the 2000s 50% of all hospitals and beds, and ‘curative visits’ in Tanzania were provided by FBOs (7). It is estimated that there are 700 hospitals and dispensaries currently under the Christian Social Services Commission in Tanzania (8).

Can FBO development aid, however, promote health improvement in recipient countries? This is ‘a topic of substantial debate’, according to Bendavid (9). The authors attempt to quantify health improvement and conclude, with a cautious yes, that life expectancy has increased and under five mortality decreased thanks to development aid. What is also clear is that, in the past, development aid has provided enormous financial inputs in health systems. In recent years, aid development projects have been under increasing scrutiny. In response to this, the 2005 Paris Declaration and the 2008 Accra Declaration (10) provided guidance on how to work in partnership in development projects. This is of importance in countries in which donors contribute considerable proportions of the overall health budgets; in Tanzania, this proportion amounts to 44% (11).

Over the past half-century, international non-governmental organisations (NGOs) have made many contributions to the health and development needs of low-income countries. ‘They have brokered funding, established programmes, conducted research and helped deliver services on an enormous scale’. However, the role of NGOs is changing, and there is a trend away ‘from managing service delivery programmes to providing more technical assistance’ (12: 915).

The focus of this paper is on an FBO-run nurse training school. In 2007, a hospital management team and I, an international health professional and researcher, started to set up a new nursing school attached to an old ‘mission hospital’ in rural Tanzania. Initially, this meant conceiving the idea with local partners, planning it, finding funding, and obtaining the necessary official permissions. The trigger to start was a meeting with the chairman of a Tanzanian church who had a plan and vision to build and run a nursing school at a mission hospital and church compound in his ecclesiastical province. Now, 9 years later, it is time to reflect and tell the story of the evolution of a nursing school in Tanzania describing the issues we confronted and how, in the future, they might be best overcome. The aim of this study was to reflect on the issues experienced whilst establishing and implementing a FBO nursing school and make recommendations for other similar initiatives.

## Methods

### Study design

I have used an auto-ethnographic approach to describe how the nursing school project evolved through different phases. Auto-ethnography is one of the newer developments in ethnographic inquiry, ‘in which the researchers’ own thoughts and perspectives from their social interactions form the central element of a study’ (13: 513). Auto-ethnography has been defined as ‘autobiographies which self-consciously explore the interplay of the introspective, personally engaged self with cultural descriptions mediated through language, history, and ethnographic explanation’ (14: 742). Auto-ethnographers ‘vary in their emphasis on the research process (*graphy*), on culture (*ethno*), and on self (*auto*)’ (p. 740).

The auto-ethnographic approach has allowed the presentation of a detailed narrative description that might otherwise be lost, for example, the emotional consequences of coping with the many ups and downs experienced (15). This exercise is useful because the project was a classical development project study with outputs, outcomes, deliverables, milestones, and so on that needed to be successfully achieved in difficult settings. The auto-ethnographic manuscript shall serve as a small-scale case study, to illustrate how several general (and potentially global) issues are deconstructed in local settings and how they can influence health development projects in developing countries.

The narrative is a personal account of the author's experience and is therefore written in the first person; it has been analysed and contextualised. This retrospective narrative is about the progress of the nursing school project (‘graphy’), and how it evolved over the phases and years. Emphasis is on the intercultural aspects (‘culture’) that are inherent in a development project of this type. Finally, it is about self (‘auto’), the author's perception and interpretation of events. This method provides the author with the space to write about himself: that is, about me as the project coordinator and how I felt, how I interpreted the difficulties, and how I analysed the evolution of the nursing school project. My professional background is nursing, public health, and epidemiology, and I was employed with the Division of Tropical and Humanitarian Medicine at Geneva University Hospitals. I was the project leader of the Nursing School Project and have been travelling to Tanzania for project follow-up two to three times a year since 2007. Since auto-ethnography is about ‘auto’, I investigated how I was and am involved in this project; the account is personal and does not reflect or represent the views of others. It is my narrative account, which I use as data for the analysis to interpret and contextualise.

In their overview on auto-ethnography, Ellis et al. (15) comment on ethical issues: Researchers do not exist in isolation, consequently ‘when we conduct and write research, we implicate others in our work’ (p. 6). These ‘relational ethics’ are heightened for auto-ethnographers. Even though I write about myself and do not judge others (project partners for example), I have anonymised colleagues, institutions, and places, to protect them and avoid any perceived discrediting. I am doing this also in view of further collaboration, because, as Ellis et al. rightly point out, ‘auto-ethnographers must stay aware of how these protective devices can influence the integrity of their research as well as how their work is interpreted and understood. Most of the time, they also have to be able to continue to live in the world of relationships in which their research is embedded after the research is completed’ (p. 7).

I submitted this manuscript to the leadership of the nursing school before publication to get feedback. Comments included the following: ‘We do not feel exposed, on the contrary. What you write, is what happened. We have no objection to it. You can go ahead’. This could be viewed as an endorsement and ‘ethical review’ from the Tanzanian partners.

### Study setting

Tanzania is an East African country with an estimated population of 48 million. Poverty is a key factor in the health of the population and in the characteristics of the health system. In 2008, Tanzania ranked 201 among 229 countries in terms of per capita of gross domestic product. Physical and human resources available to all elements of the health care system are meagre. In 2007, the Ministry of Health and Social Welfare declared a workforce crisis and set a target to increase its health resources fivefold by 2017 (16: 36).

The health system in Tanzania consists of several levels of health care facilities: national hospital, referral hospital, regional hospital, district hospital, health centre, and dispensary. There are 132 districts in Tanzania. Most of these have a government-run district hospital; others rely on FBOs to sponsor non-governmental hospitals, which become designated district hospitals and are then eligible to receive government subsidies. District hospitals offer outpatient and inpatient services not available at dispensaries or health centres, such as laboratory and X-ray diagnostic services and surgical services, including emergency obstetric care (16: 37).

The size of the health workforce (both health professionals and other health care workers) has declined both in absolute numbers and in relation to the size of the population. The decline in absolute numbers was marked during the 1990s when the Government of Tanzania retrenched the health workforce and imposed an employment freeze, resulting in a loss of one-third of the health workforce (16: 39). As in every health system, nurses account for the largest proportion of health professionals (17). As a consequence of this employment freeze, the dearth of health personnel was most pronounced among nurses. After years of structural adjustment, this trend has only been reversed recently and contributed to the re-launch of primary health care in Tanzania that aimed at equality in access to health care (18). It is interesting to see that in the 1970s, Tanzania was leading the global health agenda and influenced the debates that led to the Alma-Ata declaration (7). It also set the pace in the World Health Organization's (WHO's) new commitment to Alma-Ata revisited (19).

The district in which the nursing school project described in this paper is located is one of several in the region, comprising about 1,000 km^2^ and is on the border of a neighbouring country. Half a million people live in the district, and most of them are farmers, 21% of the population living below poverty line. The infant mortality rate in the district is 165 (compared to the Tanzanian mean of 109).

At the time of the project inception, there were two hospitals in the district: the district hospital and a mission hospital, an FBO. There were very few nurse-training facilities in the region in general. In Tanzania, there are two types of nursing schools: 1) certificate nursing schools, with the most basic training level taking 1 or 2 years (a lower level than diploma); and 2) ‘full-fledged’ diploma nursing schools, providing the full nurse training curriculum, of 3 years’ duration. In order to accelerate the output of newly trained diploma nurses, the Ministry of Health nursing curriculum (which is mandatory for all training facilities) was shortened from 4 to 3 years. However, there was no diploma nursing school at all in the district described in this study. Even today, according to a recent report on Human Resources for Health, the Primary Health Services Development Program 2007–2017, the region discussed here is under resourced in terms of nursing care (20).

This is the context in which the project took place. Using an auto-ethnographic case study design, my aim is to 1) reflect on the issues experienced whilst establishing and implementing an FBO nursing school and 2) make recommendations for others undertaking similar initiatives. There are two underlying questions: Why was providing new nurse-led services so difficult, and which factors helped the project achieve its goals?

## Results: descriptive field notes

The data are presented chronologically.

### 2006

I met the chairman of one of the Tanzanian churches. He told me about his church and the projects there, a lot about a mission hospital, and the plan to build and run a nursing school. He asked for help when he realised that I was working at the Division of Tropical and Humanitarian Medicine at the University Hospitals of Geneva and that I had been involved in health-related development projects for many years. His plea touched me, not only because it was about church-related health services (I had worked for 7 years in that field in Angola), but also because I was a lecturer at the Institute of Nursing Science (University of Basel).

### 2007

In my first trip to Tanzania, the Hospital Management Team proudly showed me around. The 150-bed hospital was old (given the long-standing presence of the respective churches in the region), clean, under-occupied, under-equipped (few essential drugs), and understaffed. There was a shortage of nurses in all wards. Although formerly, nurses often preferred mission hospitals to the governmental ones, this trend had been clearly reversed in recent years. Thanks to the so-called basket funds, the Ministry of Health and Social Welfare was able to pay decent salaries to health personnel. The Health Basket Fund was a joint mechanism between the Ministry and several donor organisations that collaborated in a joint arrangement for procurement and financial disbursement to execute the health sector plan of action. Although it was seen as an instrument for Tanzanian ownership of all activities in the health sector (21), in reality FBOs had only limited access to these funds and were not able, by themselves, to provide comparable salaries to their employees.

The team tried to persuade me to support them and their vision of their own nursing school. They took me to another hospital in a far-off village (4 h of dirt road) and showed me round the hospital and the adjacent nursing school. They succeeded in convincing me that it was feasible to set up a nursing school in a district-size hospital in a remote rural area. They explained eloquently that there was no diploma-granting nursing school in the region, neither governmental nor FBO. Back in their own region, they showed me a potential site for a future nursing school in the vast compound, which belonged to the church. On my return from Tanzania, I started writing up reports and concepts. The drafts I sent to the hospital management team remained unanswered for several months, which I found puzzling. Finally, after some emails and phone calls, the medical director of the mission hospital reassured me that the proposal for the nursing school was being reviewed favourably.

### 2008

This year's visit in Tanzania had a specific focus: plan a nursing school for the mission hospital. We were working with the hospital management team to sketch out a plan for a diploma-granting nursing school. We established the network of stakeholders, district authorities, district health officers, regional health officers, church authorities, education and accreditation institutions, other hospitals, NGOs, and ecumenical institutions. In Dar-es-Salaam, we made various visits to governmental officials, Ministry of Health officials, the Swiss embassy, and international institutions involved in health-related country wide support. Finally, we met many people in different local health organisations: researchers, teachers, policy makers, nursing school principals, directors of assistant medical officers, NGO officials, expatriates, and Tanzanian key persons, all of them were potential allies for the nursing school project. Back in Switzerland, I drew up a draft project proposal. The Tanzanian partners’ comments were enthusiastic, but those of my colleagues in Geneva were clearly less so: ‘Is it not too complicated? Why support the FBO? Is it financially feasible?’

### 2009

This year the goal was networking and ‘stakeholder-hunting’, not in Tanzania, but in Switzerland. Thanks to some funding by a Swiss organisation, the medical director of the mission hospital was able to spend a few weeks in Switzerland. This was the opportunity to write a proposal for the ‘Mission hospital Diploma Nursing School’. Well, only partly: writing ‘interculturally’ on the same document was a challenging task and took time. Between the writing time slots, we toured Switzerland to visit a number of institutions, until we met one with a leadership that was interested in embarking on a new project and encouraged us to submit a proposal.

### 2010

A visit to Tanzania early in the year helped clarify how the hospital management team intended to proceed. After a SWOT (Strengths-Weaknesses-Opportunities-Threats) analysis workshop, moderated by a Swiss colleague who accompanied me on that trip, the hospital management team was able to argue convincingly that a nursing school attached to the hospital was a valid response to various challenges. These included the lack of young staff at the hospital, the status of a rural hospital integrated in the district health plan, and an FBO's contribution to the Primary Health Services Development Program. At the end of this trip, we adjusted the project plan with church officials and the regional health officers. Back in Switzerland, I worked on the proposal as per the template provided by the Swiss organisation. The medical director of the mission hospital attended an International Global Health conference in Geneva, for discussions about who should be the project contact person on site. We agreed that it should be him, because he was the medical director of the mission hospital. We submitted the proposal, waited, received a list of necessary modifications, resubmitted the revised version, got a positive response, and were happy.

Our happiness was, however, short-lived because I then received the news that my future main counterpart in Tanzania, the medical director, had resigned from his duties. We did not get official news for several months. Whilst I was waiting, I realised that, although mobile phone networks are ubiquitous even in the remotest rural areas and have facilitated many development projects, sorting out misunderstandings, delicate issues, looming conflicts, and the concealed agendas of hidden stakeholders were still virtually impossible to resolve by these means. So I was waiting, shaking my head intermittently. I was not getting any answers, clarifications, or cues on how to interpret what had happened. Was it health politics, church politics, party struggles, personal disagreements, hospital policy? I did not know. But I needed to decide: shall we move on and start with the project? Or shall we cancel it even before it gets going, in the absence of any guarantee that the commitments of our partners in Tanzania can be relied upon, and because the one person whose name was on the project proposal was no longer a player on whom we could depend. The year ended on this note. But we did not stall the project. We decided to trust our partners and planned the kick-off meeting in early 2011.

### 2011

I travelled to Tanzania with some apprehension. What will the hospital management team look like? Will I be meeting the hospital director who resigned? How will we be perceived—as project partners or as the Swiss guys who are there to bring in money? Being there helped a lot. I was accompanied by the administrative director of a university institute. The excitement was infectious. The project management team had a new medical director (a clinical officer), the head nurse (who was to move from the hospital to the nursing school), a hospital administrator, and an accountant.

In countless meetings with different participants and stakeholders, we developed Memoranda of Understanding that addressed many issues: Who sits in the project management team? How shall we handle finances? What type of school board do we want, church officials from the head office and/or civil servants (Ministry of Health, district councils)? What about housing? There was a need for student dormitories and, according to the project management team, for staff houses. We agreed to fund student dormitories, but as for staff housing, we could not re-allocate funds that were earmarked for other items. We had to tell the team, that ‘Geneva’ would not be able to provide staff houses. The disappointment was tangible. We opened an account in a Tanzanian bank for our project funds, and I deposited the cash that I had brought with me. This time, I saw delight on the faces of the project management team.

Shortly after that kick-off meeting, I became increasingly worried. The two colleagues who were helping me set up the project had come back from their field trip with disturbing news: construction of the dormitories was slow, in spite of enormous quantities of cement bought by drawing on the newly created nursing school account. The classroom buildings were being constructed very poorly. The numerous withdrawals of funds sometimes on a daily basis raised concerns among the project partners in Switzerland. The new medical director of the school, a clinical officer (not a medical doctor) had difficulty handling the daily burdens of running a hospital and contributing to the nursing school project. A group of supervisors consisting of an accreditation council member, a Ministry of Health advisor, and a representative of the Tanzanian Nursing and Midwife Council visited the new nursing school site. The group provided a report and a list of requirements that had to be fulfilled before the institution could function as a diploma-granting nursing school. The most pressing one was that the head nurse of the hospital, designated to be the nursing school principal, did not fulfil the necessary certification for this role, and therefore had to be replaced. A further difficulty was that most of the available nursing school tutors were retired, necessitating the need for a succession plan.

Back in Switzerland, after discussions and consultations with different people (not necessarily involved in the project) we wrote a carefully worded letter to the project management team and the head office of the church. We offered different scenarios, all of which recommended postponing the start of the nursing school (the recruitment process, entry exams, enrolment of students, etc.), in order to better prepare the sites, the selection of the tutors, and the agreements with hospitals. This time, at least, I received an email with an attached letter that explained their reasoning. They flatly refused to postpone, saying: ‘Things can change quickly, we have to seize the opportunity now; otherwise we will never get going’.

Since the project was funded by governmental money (Canton of Geneva), we felt that it was not appropriate to provide funds for a project that had not received official and governmental approval. We stopped the funding. In autumn, I was invited to assist at the official launch of the nursing school (and to provide the Swiss and the Geneva flags!). Uneasy about whether to attend or not (the ‘moratorium’ was still in place), the decision was taken out of my hands: my mother had fallen severely ill and needed me to stay in Switzerland. This was how the year ended.

### 2012

I planned my annual trip to Tanzania with the apprehension I had felt in the previous year, except even worse. It was a last-chance mission. Either we would be able to unlock the project by reaching an agreement with the project management team or we would stop the funding entirely and look for other fundable projects in Tanzania. The funds were still available and earmarked for nurse training in an African country.

Two colleagues with project management experience joined me on this trip. Upon arrival in Tanzania, we were quickly taxied to the large hospitals and introduced to a large number of students: They were students from the region, doing their internships. We were amazed to see this but we were even more amazed to see how the new nurse principal was running the school. Clearly she had the required leadership skills, managed the school with assured calmness and enthusiasm that showed her experience in handling expatriates. Moreover, she had various qualifications, including a nursing education degree from the Muhimbili University of Health and Allied Sciences (22), and substantive experience that included managing infectious disease control in different areas, and working in both FBO and government health-related projects. We were equally impressed to see a Memorandum of Understanding, signed between Ministry of Health and Social Welfare and the nursing school project. This was a relief: the project would proceed.

I went to Tanzania twice again in 2012. For the first time, we were able to provide content-related input about nursing and nurse training and not just focus on management and administration. In June and November, we ran seminars on teaching methods. These trainings of trainers were for the nurse teachers, tutors, and preceptors (clinical supervisors for nursing students in hospital wards), not just at the mission hospital, but also for staff at three governmental hospitals where students were doing internships on rotation. A new problem surfaced. During the first workshop on teaching methods, participants had received a *per diem*. For the second workshop, we decided not to repeat this, since we realised that the available funds had to be prioritised for buildings. Quickly all preceptors from the hospitals other than the mission hospital stopped attending and did not return, although they were highly satisfied with the first workshop. We found ourselves in the middle of an annoying discussion of *per diems* and the disease we called *perdiemitis* (23).

Of note, at the end of 2012, we had the first ‘real’ board meeting, with board members from government (regional Ministry of Health, parliament, district council, and zonal office of health professional training) and from senior church staff, as well as from the nursing school. It was important that a lot of discussion took place about the prerequisites for the nursing school to become a diploma-granting institution, the most important being the skills lab, the computer lab, and the library. Whereas we had known this for some time, what appeared to be new was the requirement to put all of these facilities in one building. We called it the ‘lab-lab-lib’ fund, which required some considerable budget reallocations. I insisted (not for the first time) that we needed formal documentation in order to justify these budget allocations to the donors. I received no such written details, and I suspected that this kind of shifting requirement was handled flexibly whenever the funding was external and entailed increased needs and the expectations whether at the FBO level or the governmental level. What could we do if we wanted to establish a diploma-granting nursing school?

### 2013

I planned another three stays in Tanzania for 2013. To go there three times a year proved a good move that allowed me to stay close and establish rapport on site. Two workshops were carried out, thanks to a nurse practitioner who had worked internationally for many years. The topics were clinical skills and clinical assessments. For the first time since the project started, we were able to do what we intended right from the beginning: provide training for both trainers and nursing students during the same stay, as follows. First, we provided the training of trainers in theory and practice, and, second, training sessions for the nursing students, in which recently trained trainers put into practice what they had just learned by teaching the nursing students.

For the third mission this year, I travelled with an information technology specialist and a health economist. Classes were progressing with 70 students well into their second year. The lab-lab-lib building was coming along. The information technology specialist was working with a local technician to set up an Internet connection and computers on site. We grasped the opportunity to introduce tutors to computer use and assist a tutor providing computer training for the students.

The health economist coached the nursing school administration. Accounts were now run separately from the hospital administration. The student tuition fees were the main income, which generated considerable revenue for the nursing school. For the sake of transparency for the donor organisation as well the head office of the church (who had sent a new accountant to help the nursing school director manage funds), a new planning tool including a financial planning and reporting system was put in place.

This year seemed to be the smoothest project year so far. By now, the partnership with the nursing school was characterised by mutual trust, and conversations seasoned by occasional jokes and laughter were now possible. The donor organisation was even willing to continue funding after the 3-year budget, so as to support, as they put it, the consolidation of the nursing school. Two things dampened my euphoria: there was still no permission to run a diploma-granting school, and worryingly, but less palpable was a looming conflict between the nursing school and the mission hospital. Apparently, the hospital directorship, felt ‘neglected’ by the donor organisation and was jealous of the nursing school, since the project funds were earmarked for the nursing school and not the hospital. The nursing school, on the other hand, felt pressured to put services in place not only for itself, but also for the hospital, including access to Internet, WLAN, and computer facilities. We eventually succeeded in establishing relationships in the region (through ‘backdoor diplomacy’), in which all parties involved – nursing school, mission hospital, church, district health – recognised that they were in a win-win situation that was mutually beneficial.

### 2014

January brought another double pack of training of trainers and teaching of students. This time, a Swiss-based nurse expert taught wound care and dressing. Both classes for the group of tutors and the nursing students were successful. However, our aim of encouraging the hospital to participate in these courses was not working. We thought that this topic was crucial in a rural hospital setting and that teaching it would be a win-win-situation: not quite. The hospital staff attended only reluctantly or not at all; there were no *per diems* on offer. Apart from the clinical training activity, as usual there were a few planning rounds: preparing the inauguration of the school in a few months, writing an information booklet, discussing furniture and equipment of the ‘lab-lab-lib’, fine-tuning work with the constructors, having a look at financial accounts, and finalising the set-up for the computer lab. We also exchanged ideas about what areas to fund after the conclusion of the 3-year initial funding period. Hygiene, water and sanitation issues appeared to be important topics for both the nursing school and the hospital: incinerator, water supply, drainage systems, and disinfection were the likely topics for the future. It was hoped that these issues could be addressed by involving three types of professional groups: nurse students, tutors, and hospital staff. Above all, the question loomed: can this be sustainable?

The questions became even more pressing when I received phone calls from a friend in Dar-es-Salaam saying that things were getting ‘hot’ in Tanzania: the hospital director was not satisfied with the nursing school; students were not welcome at the hospital, the nurse principal was ill (because of the conflict with the hospital?), and on the verge of resigning. I experienced another fit of serious head shaking. After an intense episode over several days with many phone calls and emails between the project coordinator (myself) and church officials in Tanzania and Switzerland, contact with the director of human resources of the Tanzania Health Ministry, influential people in Geneva and Basel University, things appeared to be settling down. Fortunately, the commission charged with assessing the ability to run a diploma-granting school visited the site and provided guidance on how to start the diploma-level nurse training. We expected their report soon. Indeed, we received the document a few weeks later, with a governmental authorisation to run diploma courses.

## Results and analysis

There were three phases to the project: the preparatory phase of the nursing school project during the period from 2006 to 2010, the inception phase from 2011 to 2012, and from 2013 onwards, the implementation phase. Each phase was characterised by specific themes (Table 1).

**Table 1:** Phases and themes of the project

Phase	Themes
Preparatory phase	Motivation Communication
Inception phase	Managing the unknown Managing expectations
Implementation phase	Handling conflicts Flexible planning

### Preparatory phase: motivation and communication

*Motivation*: During the first 5 years (2006–2010), all the partners and stakeholders had to get on board. These included, first of all, the local partners in the region, church people, and Ministry of Health and Social Welfare officials; then partners in Switzerland, including potential donors, each of whom responded to different ‘cues’ or motivations. What was my own personal motivation? After years of ‘pure research’, I was thrilled to have the chance to put things into practice and work at the grass-root level.

*Communication*: Despite my years of experience in researching and implementing interpreter services in Swiss hospitals, I was surprised to realise that intercultural communication was also an issue among project partners. No knowledge of Swahili was an obstacle, mysteries and tensions remained unresolved, and the communication skills (of all of us involved) did not facilitate the resolution of sensitive matters. We did not have the linguistic skills to discuss topics like resignations, betrayals, and uneasiness about developments in the church or in local politics.

### Inception phase: managing the unknown and managing expectations

Various new themes emerged during the inception period.

*Managing the unknown*: The lack of written documents (letters, emails, scans, attachments) stressed me most of the time. I had to learn to carry on without answers to certain puzzles (cultural, communicational, political, health policy, social). Often ‘letting go’ was the only option and this frequently proved successful. Or, to put it differently: managing the unknown meant not sticking to assumptions and preconceptions. It also implied letting the local project partners negotiate without the Swiss project partners.

The unknown also came in the guise of surprises! If I had been more open to that possibility, I would have recognised earlier that our project partners did well not to follow our suggestions to postpone the opening of the nursing school, but to go ahead and reach the necessary agreements with Ministry of Health and Social Welfare. If I had been more open to letting them go ahead, I would have been able to accept their decisions (even if contrary to our own) and would have been even more pleased to discover that these turned out to be good ones.

*Managing expectations:* One of the grudges I had (and still have) is that most of the partners in Tanzania (closely or remotely involved in the project) have the misconception that there was a bottomless pit of Swiss donor funds that I could command at the snap of my fingers.

### Implementation phase: handling conflict and flexible planning

*Managing conflict*: During the 2013–2014 period, other themes emerged, above all ‘conflicts’. Could the project handle conflicts and their implications in terms of ownership (nursing school ownership, hospital ownership, and district government ownership)? Unfortunately, my assumption that the hospital would feel it was a win–win situation to have its own nursing school was too optimistic and not universally held. The hospital directorship was disappointed (my interpretation) that it did not directly benefit from the Swiss funds. This required a lot of discussions between different interest groups.

*Flexibility*: ‘Flexibility in planning’ or the revision of targets. We had to change some of them substantially. Due to the insistence of the Ministry of Health and Social Welfare that one single building should be built incorporating the skills lab, computer lab, and library, most of the available projects funds went into infrastructure. Although I was not quick to accept that the ‘lab-lab-lib’ was a good idea, I am glad I arrived at that realisation. The finalised (or at least soon-to-be finalised) building became the perfect publicity for the fledgling nursing school, showing the population and the health authorities alike that the nursing school was likely to become an important player in the region and could address the workforce shortage efficiently.

This newly acquired flexibility also helped me to see that the three areas we analysed a few years ago (primary health care, human resources for health, and the importance of public–private partnerships with FBOs) were being adequately addressed through the present nursing school project in rural Tanzania.

## Discussion

This auto-ethnographic study focused on a small-scale development project in Tanzania that intended to train nurses to be deployed to primary health care facilities services. The six themes that emerged provided a number of learning points.

*Motivation*: Motivation could be maintained against all odds because the rationale of the project was fitting. The primary health care focus and the vision of strengthening the nursing workforce and working in an FBO/public–private partnership setting helped me to carry on in the midst of uncertainties.

*Communication*: Communication is an ingredient for every (development) project. In retrospect (with auto-ethnographic eyes), I realised that I underestimated the difficulties involved in communications between partners that live separated by roughly 6,000 km.

*Managing the unknown*: I was able to learn a lot from my local partners, especially from the nurse principal about coping with the unknown and how to make the best out of it. Creative problem solving is one of the 10 areas in which developed countries have the most to learn from developing countries (24). I can confirm and was frequently surprised by this.

*Managing expectations*: Both my experience and the literature show that managing expectations is a common topic in development projects. In fact, documents like the Accra agenda for action (9) address exactly how project partners can mutually determine what is to be expected and what is to be provided.

*Handling conflicts*: Almost all themes, which emerged over the course of the project, have to do with handling conflicts. With so many participants with agendas (some of them hidden), tensions were unavoidable, although the amount of money involved was relatively small compared to other health development projects (550,000 $ over 3 years).

*Flexible planning and revising targets*: An important lesson learnt is the ability to navigate between the agendas of the different people involved and to be aware of the topics that are sensitive in the current health landscape. Hot topics include incentives, the financial struggles of district hospitals, poor awareness of primary health care, churches’ interests in embarking on health projects, and the decentralisation of health services. Taken together, these topics mean that a development project like the nursing school needs to proceed very carefully and in accordance with the agendas of all three levels of health policy – at national, regional, and, above all, at the district level.

Although the study took an auto-ethnographic approach, the nursing school directorship highlighted that the themes and lessons of this work raise questions for others that might be helpful for them in the future.

Such a study with its narrative and qualitative design necessarily has limitations. I chose to focus on one single project, instead of providing a broad overview of successful or unsuccessful development projects. This account has been condensed.

Was it good to use an auto-ethnographic design? When Delamont lists her arguments against auto-ethnography, some of them apply partly to my study: It is experiential not analytic; it focuses on the wrong side of the power divide; it abrogates our duty to go out and collect data; ‘finally and most importantly “we” are not sufficiently noteworthy to be published in journals, to teach about or to draw attention to ourselves. The important questions are not about the personal anguish (and most auto-ethnography is about anguish)’ (25: 2). Auto-ethnography is about ‘highly personalized accounts where authors draw on their own experiences to extend understanding’ (26) and appears to be the best possible method suitable to reflect on the issues experienced whilst establishing an FBO nursing school within a rural primary health care programme.

Furthermore, this methodological design prevented me from venturing into value judgements, assumptions, or accusations. This, in turn, created the advantage that I was better able to see the real changes and achievements of the project. These were often not thanks to me, but to the merit of the Tanzanians in charge, notably the nurse principal. This approach is probably the only way to act (and write) ethically and responsibly. Otherwise, the risk of becoming paternalistic would be too great.

Regarding the potential power divide (25): Since I was the project coordinator, I represented some power and access to funding, but in fact I had neither. Contrary to appearances, especially in the eyes of health or church officials only loosely involved in the project, I had little power in decision-making. The decision-making lines of authority were diluted: different organisations had influence, including their own overt and hidden agendas. These included the FBO, the hospital, the nursing school directory board, and the church hierarchy. In addition, there was the government, including the Ministry of Health at the – local, district, regional, and national levels; the district council; and other boards such as the Nurse and Midwife Council and an Accreditation Council. In the midst of this web of different agendas, my main role was a mediating one.

## Conclusions

The project could have failed on many occasions, as shown in this narrative, and there were many stumbling blocks. Why did it not fail? From this experience, my key lessons for others would include the following:

First, find the right ratio of stakeholders, participants, and agendas, but do not overload. If you overload the project with too many participants, including stakeholders (that you cannot move on without losing one), too many friends (whom you might disappoint), and with considerably heterogeneous project partners and their hidden agendas, you are likely to fail in your project.

Second, be alert and communicate as much as possible with the staff. Do not ignore issues hoping they will resolve themselves. You risk ignoring many aspects, including the governmental organisations’ contexts, the NGO contexts, the FBO interests, the administrative challenges, the omnipresent risk of corruption, and the power of certain institutions. Finally, more generally, there are simple the barriers of culture (27), language (28), and beliefs (29). Furthermore, do not ignore change, for example, the fact that counterparts may change; nurses obtain new assignments, doctors obtain better positions and move on, staff retire, political parties move in or out of power, church chairmen are elected and ousted. Ignore any of these factors, and you set yourself up for failure.

Third, think flexibly. Do not stubbornly stick to original plans that might not be working. If one sticks stubbornly to expectations (yours or those of others), or to the power of the written word, proposal and reports, political correctness, or control, one is likely to fail.

Fourth, be realistic, do not romanticise. If you romanticise language and culture, or history (yours, the project's country, and the donors’ country), and the importance of your project, you are also likely to fail.

In retrospect I realise that embarking on such a project was a timely response to the Tanzanian's government call for strengthening Primary Health Care and rapidly accelerating the training of nurses able to work in rural areas. The most powerful motivation to continue was the shared overall vision between the Tanzanian and Swiss project partners, the shared objectives and goals and an indomitable spirit. This allowed us to continue in the face of many challenges and adversity despite the various factors that threatened to undermine the very fabric of the project and cause failure. We have not fallen prey to these threats so far. The nursing school is up and running, and our primary objective has been achieved. Of course, the future is uncertain, but with what we have achieved so far, we have started and continue to improve the provision of nursing care to Tanzanians in rural areas.

## Paper context

In 2007, the Tanzanian government called for improvements in its Primary Health Care services. Part of this initiative was to accelerate nurse training. This study reflects on the issues experienced whilst establishing an FBO nursing school and makes four recommendations for similar initiatives: First, find the right ratio of stakeholders, participants and agendas. Be alert and communicate as much as possible with staff. Think flexibly; do not stubbornly stick to original plans. Be realistic: do not romanticise.
